# Infarct size by cardiovascular magnetic resonance with delay enhancement as prognostic factor in the coronary artery disease: preliminary study

**DOI:** 10.1186/1532-429X-11-S1-P137

**Published:** 2009-01-28

**Authors:** Leticia Castellanos Cainas, Sandra Graciela Rosales Uvera, Jaime Galindo Uribe, Jorge Vazquez La Madrid, Jorge Oseguera Moguel, Florencia Vargas Vorackova, Martha Morelos Guzman

**Affiliations:** National Institute of the Medical Science and Nutrition Salvador Zubiran, Mexico D.F., Mexico

**Keywords:** Cardiovascular Magnetic Resonance, Ischemic Heart Disease, Infarct Size, Volume Index, Steady State Free Precession

## Introduction

The magnetic resonance (MR) has taken nowadays a crucial role in the evaluation of ischemic heart disease, which is regarded as the technique of reference for the assessment of myocardial viability. Delay enhanced cardiovascular magnetic resonance (DE-CMR) is a specific marker of myocardial necrosis, using this technique can determine the presence of infarct localization, size and transmurality, parameters of great importance for determining treatment and prognosis.

## Purpose

To determine if infarct size measured by DE-CMR is a prognostic factor for mortality in patients with ischemic heart disease.

## Methods

Sixty eight patients were referred to cardiovascular magnetic resonance because of suspicion or knowledge of ischemic heart disease between September 2004 and September 2008. CMR imaging was performed using GE 1.5 T system. Steady state free precession (SSFP) cine MR images were acquired in long and short axis orientation. Evaluation of functional parameters including end diastolic volume (EDV), end systolic volume (ESV), left ventricle ejection fraction (LVEF) and systolic volume (SV) indexed a body surface area (BSA).

## Results

**Figure 1:**
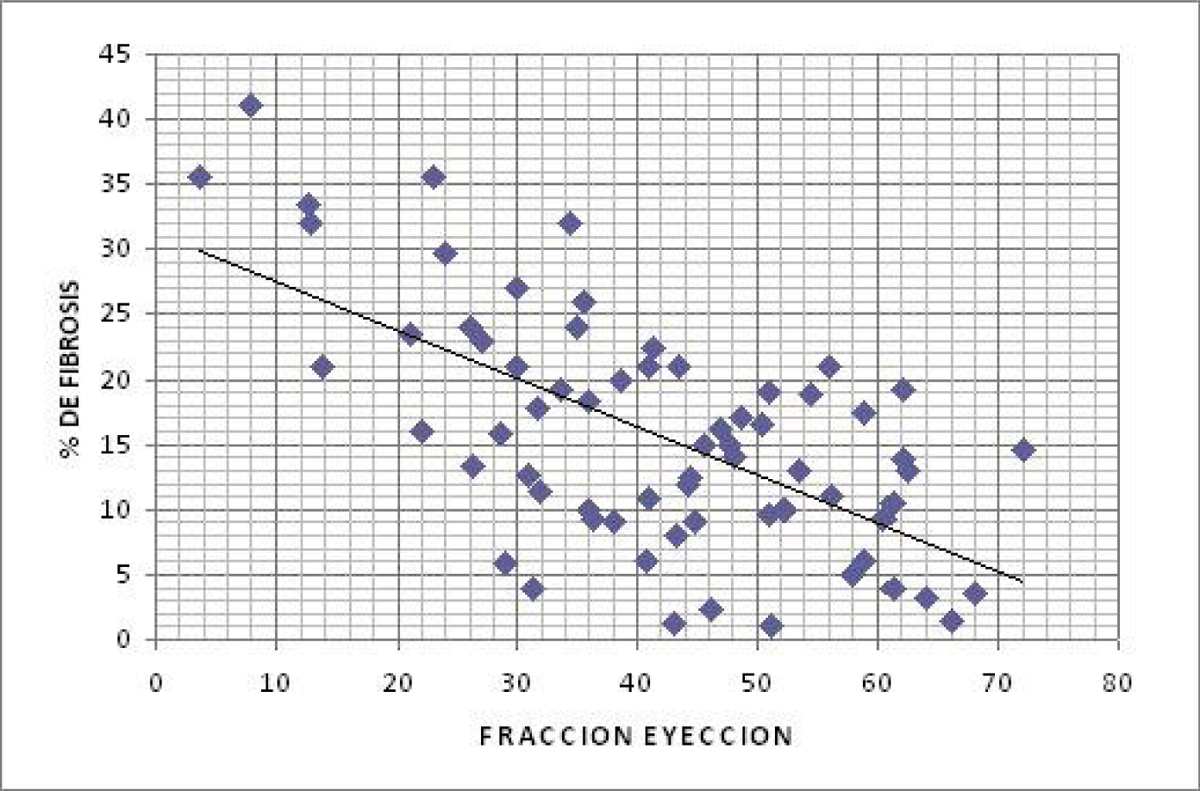
Figure 1

The average age of the study population was 65.6 (+/- 10.7 SD) years, 12% patients were in functional class III-IV NYHA, the mortality rate was 16.1%. We evaluate 1156 segments and the 39.7% of this presented delay enhancement. The myocardial infarction size was significantly higher in patients who died (21.6% vs. 14.4% p = 0.01). The relationship between infarct size, end-sistolic volume index, end-diastolic volume index, ejection fraction of left ventricle and systolic volume index, were statically significative (p < 0.001). The mayor adverse cardiac events (MACE) were presented in 90% of the group of death patients (p = 0.001). Figure 1.

## Conclusion

Infarct size measured by delay enhanced cardiovascular magnetic resonance (DE-CMR) is a prognostic factor for mortality in patients with ischemic heart disease.

